# Cardiovascular Outcomes Associated With Biologic Therapy in Inflammatory Dermatologic Diseases: A Systematic Review

**DOI:** 10.7759/cureus.103223

**Published:** 2026-02-08

**Authors:** Mohamed Eghleilib, Muaad Eghlileb, Ahmed Eghlileb

**Affiliations:** 1 Internal Medicine, Sheffield Teaching Hospitals NHS Foundation Trust, Sheffield, GBR; 2 General Medicine, University Hospital of Wales, Cardiff, GBR; 3 Dermatology, Habib Medical Group - Al Suwaidi Hospital, Riyadh, SAU

**Keywords:** atherosclerotic inflammation, biologic therapy, cardiovascular outcomes, cytokine inhibitors, dermatologic disease, hidradenitis suppurativa, major adverse cardiovascular events, psoriasis biologics, systemic inflammation, tnf-alpha inhibitors

## Abstract

Chronic systemic inflammation is characteristic of many inflammatory dermatoses, with psoriasis showing the closest association. This inflammation is closely linked to atherosclerosis and adverse cardiovascular events. Given that the same pro-inflammatory cytokines targeted by biologic therapies in skin disease are also central to cardiovascular pathophysiology, these treatments may attenuate vascular inflammation with potential implications for cardiovascular risk. This systematic review provides a qualitative analysis of the evidence on the cardiovascular effects of biologic therapies in dermatological diseases and explores potential underlying mechanisms.

A systematic search of PubMed, Embase, and the Cochrane Library identified studies published between 2005 and 2025. Using the population, intervention, comparator, outcomes, and study (PICOS) design framework, studies of patients with psoriasis, psoriatic arthritis, hidradenitis suppurativa, and atopic dermatitis treated with biologics targeting tumor necrosis factor-α (TNF-α), interleukin (IL)-12/23, IL-17, IL-23, or IL-4/13 were included. Two reviewers independently screened and extracted data. Included study designs were randomized controlled trials (RCTs), post hoc phase III analyses, population-based cohorts, registry studies, and mechanistic imaging or biomarker studies. Outcomes included major adverse cardiovascular events (MACE) and surrogate markers, including C-reactive protein (CRP), lipid indices, arterial stiffness, carotid intima-media thickness, and vascular inflammation assessed by fluorine-18 fluorodeoxyglucose positron emission tomography/computed tomography. Due to heterogeneity, findings were synthesized narratively.

Seventy-four studies were included in the qualitative synthesis, comprising RCTs, population-based cohort and registry studies, post-hoc analyses of phase III trials, and mechanistic imaging and biomarker studies. Evidence was most robust for TNF-α inhibitors, with multiple large observational studies and meta-analyses suggesting a reduced risk of MACE compared with conventional systemic or topical therapies, despite reports of worse outcomes with high-dose TNF-α inhibition in patients with pre-existing moderate-to-severe heart failure. Mechanistic studies consistently showed improvements in surrogate cardiovascular markers, including CRP, endothelial function, arterial stiffness, and vascular inflammation. For IL-12/23, IL-17, IL-23, and IL-4/13 inhibitors, available data, predominantly from pooled safety analyses, short-term randomized trials, and mechanistic studies, suggested overall cardiovascular safety but no consistent reduction in MACE. Pharmacovigilance analyses identified a potential safety signal for the IL-23 inhibitor risankizumab, with a disproportionately higher incidence of cerebrovascular events than with other biologic therapies for plaque psoriasis.

Biologic therapies, particularly TNF-α inhibitors, demonstrate potential cardiovascular benefit in inflammatory dermatologic diseases. However, results across classes remain inconsistent, reflecting heterogeneity in study design and limited long-term follow-up, particularly for newer classes. Mechanistic and clinical evidence suggest that systemic inflammation control may underpin cardiovascular improvement, yet this effect is unlikely to be uniform across biologic classes.

## Introduction and background

Chronic systemic inflammation is characteristic of many inflammatory skin conditions, with the strongest association documented for psoriasis [1,2]. Although chronic inflammation is present in other inflammatory skin conditions, such as atopic dermatitis (AD) and hidradenitis suppurativa (HS), the nature of this inflammation remains poorly understood, reflecting the limited use of targeted anti-inflammatory therapy [3,4].

As our understanding of the pathways that underpin inflammatory conditions has advanced, therapeutics have evolved from topical and conventional systemic therapies to more advanced targeted biologics [5,6]. Biologic therapy refers to the use of therapeutic agents derived from living organisms (or their components, e.g., proteins/antibodies) to modulate specific molecular pathways involved in disease [7]. The table below highlights the evolution of biologic therapy in dermatology, showing the key cytokine inflammatory mediators targeted and the conditions for which they are used (Table 1).

**Table 1:** Timeline of biologic therapies in dermatology Approximate timeline of biologic therapy development in dermatology, showing key cytokine targets, primary dermatologic indications, and representative agents. Cytokine key: TNF-α: tumor necrosis factor-α, IL: interleukin, TSLP: thymic stromal lymphopoietin

Approximate timeline	Biologic class (by cytokine target)	Main dermatologic indications	Example(s)
Early 2000s	TNF-α inhibitors	Psoriasis, HS	Etanercept, infliximab, adalimumab
Late 2000s	IL-12/23 inhibitors	Psoriasis	Ustekinumab
Mid-2010s	IL-17 inhibitors	Psoriasis, psoriatic arthritis	Secukinumab, ixekizumab
Late 2010s	IL-23 inhibitors	Psoriasis	Guselkumab, risankizumab
Late 2010s-2020s	IL-4/IL-13 inhibitors	AD	Dupilumab
2020s onward	Next generation cytokine blockers (IL-31, IL-36, TSLP)	AD, pustular psoriasis	Nemolizumab, spesolimab

The chronic inflammation underpinning dermatological conditions such as psoriasis is closely linked to atherosclerosis and adverse cardiovascular events [1,2,8]. The same pro-inflammatory cytokines targeted by biologic therapies in skin disease are also central to cardiovascular pathophysiology, suggesting that these treatments may attenuate vascular inflammation and reduce cardiovascular risk [1,2]. Despite growing recognition of this association, the comparative impact of biologics on cardiovascular outcomes (relative to conventional systemic or topical therapies) remains incompletely understood [9]. This review, therefore, examines the evidence surrounding the cardiovascular effects of biologic therapies in dermatological disease, exploring proposed mechanistic pathways linking immune modulation to cardiovascular outcomes. By doing so, it aims to clarify the complex interplay between cutaneous and cardiovascular inflammation to inform clinical decision-making and risk stratification. Notably, the review focuses exclusively on cardiovascular outcomes and systemic inflammatory modulation, rather than the cutaneous efficacy of biologics, which has been extensively addressed elsewhere [10,11].

The literature predominantly focuses on psoriasis and psoriatic arthritis, with minimal evidence available for AD and HS [12]. No significant studies were identified assessing cardiovascular outcomes, inflammatory markers, or risk factors in patients with other inflammatory dermatoses treated with biologic therapies. The scarcity of data in AD and HS, and the near absence of data for other conditions, reflect our understanding of the pathophysiology of each condition [3,13,14]. As these disorders are generally understood to confer lower cardiovascular risk compared with psoriasis, biologics are seldom first-line treatments, and research has largely prioritized cutaneous efficacy over cardiovascular endpoints such as myocardial infarction and stroke [14-16]. Additionally, the rarity of some conditions, such as pemphigus vulgaris, poses challenges for large-scale investigation. Although this may indicate an incomplete understanding of these conditions and their systemic implications, the potential of biologics in these patient groups remains beyond the scope of this systematic review.

This review evaluates the cardiovascular effects of biologic therapies across the main drug classes: TNF-α inhibitors, IL-12/23 inhibitors, IL-17 inhibitors, IL-23 inhibitors, and IL-4/IL-13 inhibitors. Emerging agents such as nemolizumab and spesolimab were excluded, as they are not yet widely established in clinical practice and have only recently received regulatory approval for limited indications: nemolizumab for AD and spesolimab for acute generalized pustular psoriasis [17,18]. These conditions are relatively uncommon compared with chronic plaque psoriasis and AD, and no large-scale cohort studies or randomized trials currently assess their cardiovascular outcomes. Their inclusion would therefore introduce speculation rather than evidence-based analysis. Nonetheless, for the established biologic classes, this review examines the available evidence regarding cardiovascular events, inflammatory biomarkers, subclinical atherosclerosis, and metabolic parameters, while also addressing reported adverse effects, safety concerns, and methodological limitations. Given the predominance of research on psoriasis and psoriatic arthritis, this is reflected proportionately in the discussion.

## Review

Studies were selected according to predefined inclusion and exclusion criteria based on the population, intervention, comparator, outcomes, and study design (PICOS framework). The criteria were established in advance to ensure that only relevant studies were included, examining cardiovascular outcomes associated with biologic therapy in autoimmune dermatologic diseases. Eligible studies were limited to English-language human research published between 2005 and 2025. The detailed inclusion and exclusion criteria are summarized in Table 2.

**Table 2:** PICOS criteria Eligibility criteria for study inclusion and exclusion based on the PICOS framework. PICOS: population, intervention, comparator, outcomes, and study design, TNF-α: tumor necrosis factor-α, IL: interleukin, CRP: C-reactive protein, AD: atopic dermatitis, HS: hidradenitis suppurativa, HF: heart failure

Category	Inclusion criteria	Exclusion criteria
Population	Adults (≥18 years) diagnosed with autoimmune dermatologic diseases, including psoriasis, HS, AD, pemphigus vulgaris, and bullous pemphigoid.	Studies focusing exclusively on non-dermatologic autoimmune diseases (e.g., rheumatoid arthritis, inflammatory bowel disease).
Intervention	Biologic therapies used in dermatology practice include TNF-α inhibitors, IL-12/23 inhibitors, IL-17 inhibitors, and IL-4/13 inhibitors.	Non-biologic immunomodulators used alone without a biologic comparator.
Comparator	Conventional systemic treatments (e.g., methotrexate, cyclosporine), placebo, or no treatment.	—
Outcomes	Primary: Incidence of cardiovascular events (myocardial infarction, stroke, cardiovascular death, HF). Secondary: Surrogate cardiovascular markers, including CRP, carotid intima-media thickness, and lipid profile.	Studies without extractable or clearly defined cardiovascular outcomes.
Study design	Randomised controlled trials, prospective or retrospective cohort studies, case-control studies, and systematic reviews/meta-analyses of these study designs.	Case reports, editorials, expert opinions, conference abstracts, and animal or in vitro studies.
Language	English.	Non-English publications.
Publication window	Studies published between January 2005 and December 2025.	Studies published outside the specified time window.

Data sources and search strategy

A comprehensive literature search was conducted in three electronic databases: PubMed (24 July 2025), Embase (8 August 2025), and the Cochrane Library (8 August 2025) to identify studies evaluating the cardiovascular effects of biologic therapy in autoimmune dermatologic diseases. The search strategy combined controlled vocabulary (MeSH/Emtree) and free-text terms relating to dermatologic conditions, biologic therapies, and cardiovascular outcomes. Searches were limited to English-language human studies published between 2005 and 2025. The complete search strings and applied filters are summarized in Table 3.

**Table 3:** Search strategy Databases searched, search strategy, and applied filters were in accordance with PRISMA 2020 recommendations. The same core Boolean structure was applied across all databases (PubMed, Embase, and Cochrane via Ovid), with syntax adapted to each platform’s indexing system (MeSH, Emtree, and Cochrane/Ovid field tags). MeSH: Medical Subject Headings, PRISMA: Preferred Reporting Items for Systematic Reviews and Meta-Analyses

Database	Search strategy (Boolean terms and MeSH)	Limits and filters applied	Date of search
PubMed (MEDLINE)	(“Psoriasis”[Mesh] OR psoriasis[tiab] OR “hidradenitis suppurativa”[tiab] OR “atopic dermatitis”[tiab] OR eczema[tiab] OR pemphigus[tiab] OR “bullous pemphigoid”[tiab]) AND (“Biological Products”[Mesh] OR biologic*[tiab] OR “TNF inhibitor”[tiab] OR adalimumab[tiab] OR infliximab[tiab] OR etanercept[tiab] OR ustekinumab[tiab] OR secukinumab[tiab] OR dupilumab[tiab] OR “IL-17 inhibitor”[tiab] OR “IL-23 inhibitor”[tiab]) AND (“Cardiovascular Diseases”[Mesh] OR cardiovascular[tiab] OR “myocardial infarction”[tiab] OR stroke[tiab] OR “cardiovascular mortality”[tiab] OR atherosclerosis[tiab] OR “heart failure”[tiab] OR “carotid intima media thickness”[tiab] OR “endothelial function”[tiab] OR CRP[tiab] OR “coronary calcium”[tiab])	English language; human studies; publication years 2005–2025	24 July 2025
Embase (Ovid)	Same Boolean structure as PubMed, adapted to Emtree terms and field tags	English; human studies; publication years 2005–2025	8 August 2025
Cochrane Library (CENTRAL via Ovid)	Same Boolean structure as PubMed and Embase, applied using Ovid’s Cochrane Database syntax	English; trials only; 2005–2025	8 August 2025

Study selection and screening

**Figure 1:**
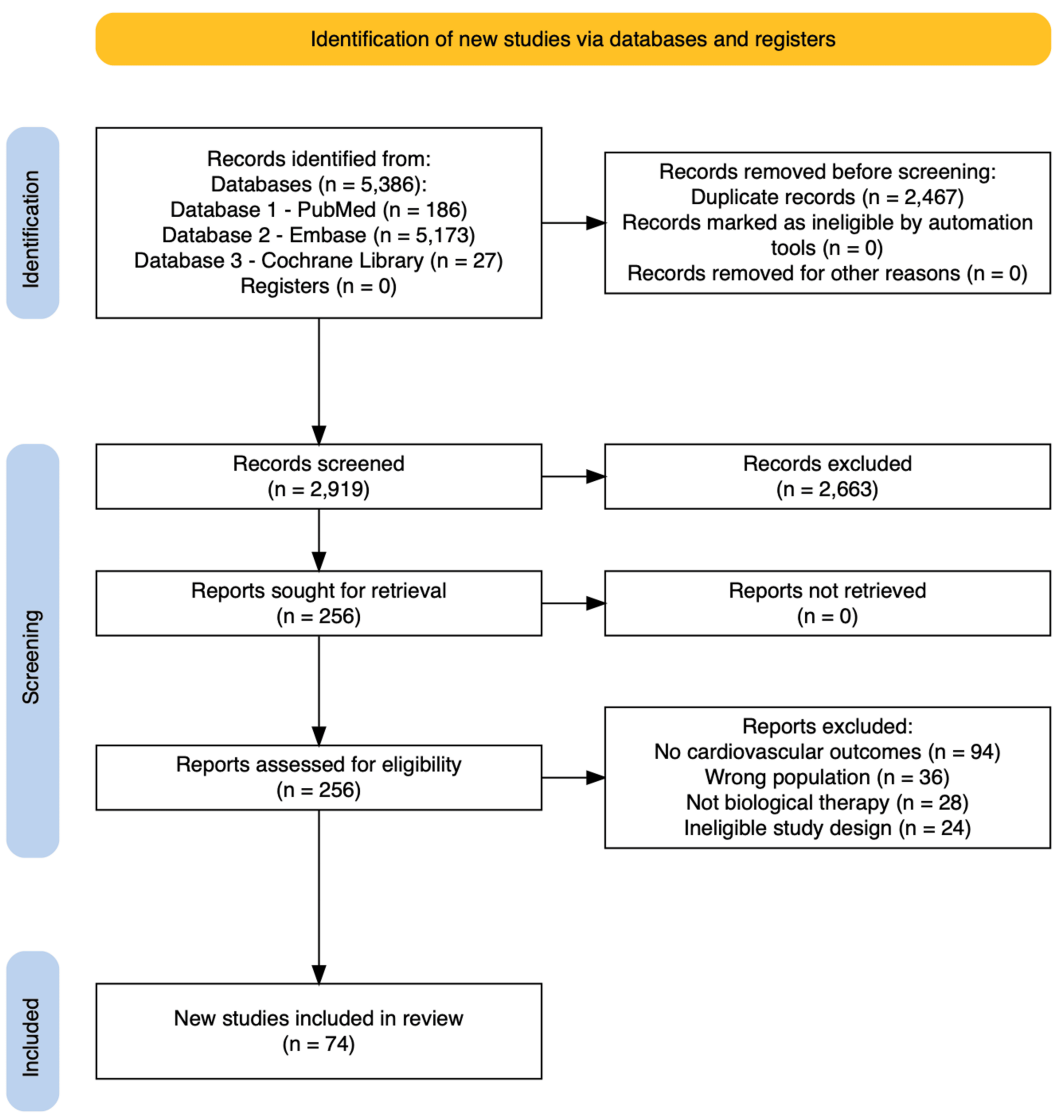
PRISMA 2020 flow diagram of study selection Flow diagram illustrating the identification, screening, eligibility assessment, and inclusion of studies in this systematic review in accordance with PRISMA 2020 guidelines. Records were identified through database searches of PubMed, Embase, and the Cochrane Library. After removing duplicates, records were screened by title and abstract, followed by a full-text assessment for eligibility. Full-text articles were excluded for predefined reasons, and the remaining studies were included in the qualitative synthesis. PRISMA: Preferred Reporting Items for Systematic Reviews and Meta-Analyses

All identified records were imported into reference management software, and duplicate entries were removed prior to screening. The initial search yielded 5,386 records: 186 from PubMed, 5,173 from Embase, and 27 from the Cochrane Library. After removal of duplicates, 2,919 unique studies remained and were screened by title and abstract against the predefined eligibility criteria (Table 2). Following this stage, 256 articles were retrieved for full-text review to assess relevance and methodological adequacy. After detailed evaluation, 74 studies met the inclusion criteria and were included in the final qualitative synthesis. The full study selection process is summarized in the PRISMA 2020 flow diagram (Figure 1). No study registries or trial registers were searched.

Data extraction

Titles and abstracts identified through the database search were screened independently by two reviewers. Full texts of potentially eligible studies were then retrieved and reviewed in detail. Data extraction was subsequently performed independently by both reviewers using a standardized form developed for this review, with each reviewer cross-checking the other’s entries for accuracy and completeness. Extracted information included study characteristics (first author, year of publication, country, study design, and sample size), participant demographics, dermatologic diagnosis, biologic agent evaluated, comparator intervention, duration of follow-up, and cardiovascular outcomes reported. Outcomes were categorized as either clinical cardiovascular events (myocardial infarction, stroke, heart failure (HF), cardiovascular death) or surrogate cardiovascular markers (e.g., CRP, lipid profile, carotid intima-media thickness, endothelial function). Any discrepancies identified were resolved through discussion and consensus. Where multiple publications reported on the same cohort, the most comprehensive or most recent dataset was included.

Data items

The primary outcome was the incidence of cardiovascular events, including myocardial infarction, stroke, HF, and cardiovascular mortality, among patients receiving biologic therapy compared with conventional systemic therapy, placebo, or no treatment. Secondary outcomes included changes in surrogate cardiovascular markers, such as systemic inflammatory markers (C-reactive protein (CRP) and erythrocyte sedimentation rate (ESR)), lipid levels, carotid intima-media thickness, and measures of endothelial function. Additional descriptive variables, including duration of dermatologic disease, comorbidity burden, and concurrent cardiovascular medications, were extracted where reported.

Risk of bias and quality assessment

Risk of bias was assessed in a structured manner across included studies, with consideration of key domains relevant to heterogeneous evidence syntheses, including selection bias, confounding, and outcome assessment. Given the diversity of study designs and outcome measures, formal scoring using a single risk-of-bias tool across all studies was not considered appropriate. Instead, methodological appraisal was informed by principles derived from the Cochrane Risk of Bias 2 (RoB 2) tool for randomized controlled trials (RCTs) and the Newcastle-Ottawa Scale (NOS) for observational studies, applied at the level of study design.

Observational and registry-based studies were recognized as more susceptible to selection bias and residual confounding than randomized trials, whereas outcome assessment was generally robust across study types. No study was excluded based on risk of bias alone; however, these assessments informed the narrative synthesis and interpretation of comparative findings. A summary of risk-of-bias domains by study design is provided in Table 4.

**Table 4:** Summary risk-of-bias assessment by study design Qualitative summary of risk-of-bias domains across included studies by study design, informed by principles from RoB 2 and NOS. Domains assessed included selection bias, confounding, and outcome assessment. RoB 2: Cochrane Risk of Bias 2, NOS: Newcastle-Ottawa Scale

Study design	Selection bias	Confounding	Outcome assessment	Overall risk of bias
Randomised controlled trials	Low	Low	Low	Low
Post-hoc analyses of phase III trials	Low–moderate	Moderate	Low	Moderate
Prospective observational cohort studies	Moderate	Moderate–high	Low–moderate	Moderate
Retrospective cohort and registry studies	Moderate	High	Moderate	Moderate–high
Mechanistic imaging and biomarker studies	Moderate	Moderate	Low	Moderate

Data synthesis

A quantitative meta-analysis was conducted for prespecified cardiovascular outcomes, including major adverse cardiovascular events (MACE), myocardial infarction, stroke, cardiovascular mortality, and selected surrogate markers of cardiovascular risk (e.g., CRP, vascular imaging parameters, and arterial stiffness measures). However, a meta-analysis was not conducted due to substantial methodological heterogeneity among the included studies. This included wide variation in outcome definitions (particularly for composite MACE endpoints), follow-up durations ranging from short-term mechanistic studies (12-24 weeks) to long-term registry-based cohorts (several years), and heterogeneity in comparator groups (placebo, conventional systemic therapy, phototherapy, or no treatment). Additionally, studies differed markedly in design (randomized trials, post-hoc analyses, observational cohorts, and registries) and frequently reported outcomes using incompatible effect measures, precluding meaningful statistical pooling.

Accordingly, a qualitative (narrative) synthesis was undertaken. Findings were grouped by biologic drug class, namely TNF-α inhibitors, IL-12/23 inhibitors, IL-17 inhibitors, IL-23 inhibitors, and IL-4/13 inhibitors, and summarized with respect to both clinical cardiovascular outcomes and surrogate markers of cardiovascular health. Within each category, the direction and strength of associations were compared descriptively, with consideration of mechanistic plausibility, consistency across studies, and potential confounding factors, including baseline cardiovascular risk, disease severity, and concomitant therapies.

Ethical considerations

This review analyzed data from previously published studies and did not involve any human participants or patient-identifiable information. Therefore, formal ethical approval and informed consent were not required.

Reporting standards

This systematic review was conducted and reported in accordance with PRISMA 2020 guidelines. The completed PRISMA 2020 checklist is available in the supplementary material. This review followed methodological guidance outlined in the Cochrane Handbook for Systematic Reviews of Interventions.

Registration and protocol

A review protocol outlining the research question, eligibility criteria, database search strategy, prespecified outcomes, and planned narrative synthesis was developed prior to commencement of data extraction and analysis. This protocol-guided study selection, outcome assessment, and synthesis were not modified in response to study findings.

The protocol was not prospectively registered with PROSPERO. This was an unfunded, investigator-initiated project conducted independently by the authors. The absence of prospective registration does not imply post-hoc outcome selection, as all outcomes and synthesis methods were defined in advance, prior to data extraction and analysis.

Funding

This research received no external funding. All work was undertaken independently by the authors as part of their academic and professional development. No financial support, sponsorship, or grants were obtained from any commercial or institutional sources.

Conflicts of interest

The authors declare no conflicts of interest related to this work. None of the authors has any financial or personal relationships that could have influenced the design, conduct, or reporting of this systematic review.

Data availability

All data supporting the findings of this study are included within the article and its supplementary materials. No additional unpublished data were generated or analyzed. Further details of the search strategy and extracted datasets are available from the corresponding author upon reasonable request.

Summary of study characteristics

A total of 74 studies met the inclusion criteria and were included in the final synthesis. Most studies evaluated patients with psoriasis or psoriatic arthritis, with smaller numbers examining HS and AD. The evidence base comprised RCTs, large population-based cohort studies, post-hoc analyses of phase III clinical trials, mechanistic imaging studies, and smaller biomarker-focused cohorts. Follow-up durations ranged from 12 to 24 weeks in mechanistic and phase IV studies to several years in registry-based cohorts. Across biologic classes targeting TNF-α, IL-12/23, IL-17, IL-23, and IL-4/IL-13, cardiovascular outcomes included both MACE (myocardial infarction, stroke, HF, and cardiovascular death) and surrogate markers such as CRP, other systemic inflammatory indices, lipid parameters, arterial stiffness, carotid intima-media thickness, and vascular inflammation assessed by fluorine-18 fluorodeoxyglucose positron emission tomography/computed tomography (18F-FDG PET/CT). The aggregated characteristics of the included studies, grouped by biologic class, are summarized in the Appendices.

TNF-α inhibitors

*TNF-α Inhibitors and Psoriasis*****

**Figure 2:**
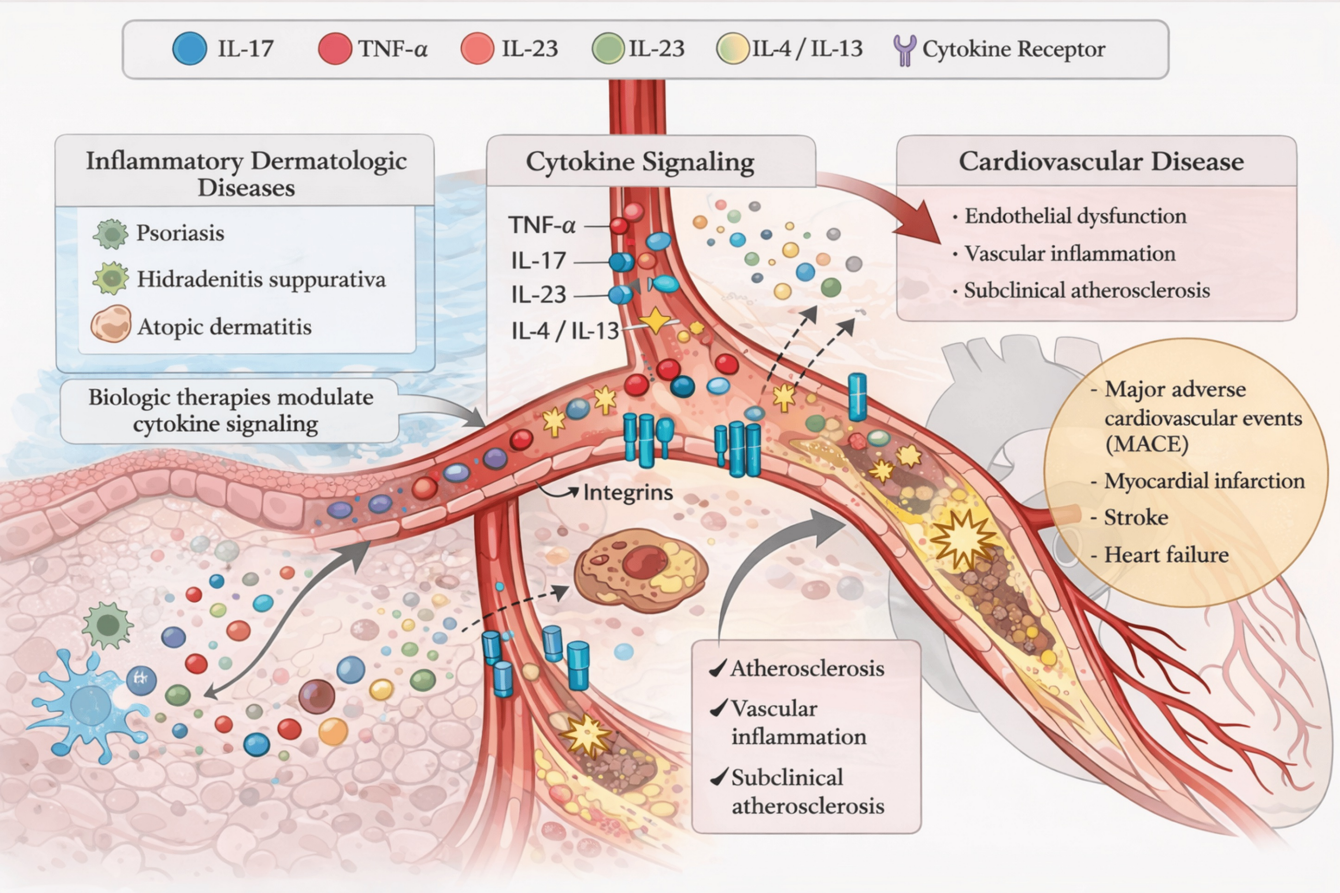
Proposed mechanistic links between inflammatory dermatologic disease, cytokine signaling, vascular inflammation, and cardiovascular disease Inflammatory dermatoses, including psoriasis, HS, and AD, are associated with systemic activation of pro-inflammatory cytokine pathways, including TNF-α, IL-17, IL-23, and IL-4/13. Modulation of these cytokine pathways by biologic therapies may influence endothelial dysfunction, vascular inflammation, and downstream cardiovascular outcomes. TNF-α: tumor necrosis factor-α, IL: interleukin, AD: atopic dermatitis, HS: hidradenitis suppurativa Image Credit: Authors. Created using ChatGPT (OpenAI, San Francisco, CA, USA) for the conceptual design and visual generation. The figure was reviewed, edited, and validated by the authors.

Figure 2 summarizes the proposed mechanistic links between systemic cytokine signaling in inflammatory dermatologic disease and downstream vascular inflammation and cardiovascular outcomes. TNF-α inhibitors were the first biologic class approved for dermatological use, namely in psoriasis and psoriatic arthritis, and remain the most extensively studied given their long-standing clinical application and the well-established association between psoriasis and cardiovascular disease [19,20]. Atherosclerosis, the primary underlying process of cardiovascular disease, is fundamentally an inflammatory process driven by cytokines and immune mediators shared with psoriatic inflammation [21,22]. Patients with moderate-to-severe psoriasis demonstrate elevated levels of CRP, IL-6, and TNF-α (biomarkers strongly associated with atherosclerotic burden and adverse cardiovascular outcomes) [23,24]. Moreover, subclinical atherosclerosis, as measured by carotid intima-media thickness, arterial stiffness, and coronary artery calcium scores, is significantly more prevalent in psoriatic populations compared to age-matched controls [24-27]. Thus, the rationale for cardiovascular protection with TNF-α inhibition is mechanistically compelling.

Multiple large-scale observational studies and meta-analyses have examined the impact of TNF-α inhibitors on MACE in psoriatic disease [9,28]. In one of the largest studies, a nationwide, population-based cohort study in Korea reported a reduced risk of MACE with biologic therapy compared with conventional systemic treatments, although the magnitude of benefit varied by agent and patient subgroup [29]. In an observational cohort study comparing TNF-α inhibitors with phototherapy in patients with psoriasis, TNF-α inhibitors were associated with lower rates of MACE over 24 months, with a statistically significant difference after only six months [30]. More broadly, in a meta-analysis of TNF-α inhibitors in psoriasis and psoriatic arthritis, TNF-α inhibitors were associated with a 33% reduction in MACE compared with methotrexate and a 42% reduction compared with topical/phototherapy [31]. Heterogeneity among studies in this meta-analysis was notable and likely driven by multiple confounding variables. For instance, in a global population study specifically examining statin-treated psoriasis patients, biologics, including TNF-α inhibitors, were reported to reduce adverse cardiovascular outcomes, suggesting a synergistic benefit compared with conventional therapies [32]. This highlights a key limitation of meta-analyses: methodological heterogeneity and insufficient adjustment for confounding variables limit the ability to disentangle treatment effects from underlying risk factors and confounding variables. Nevertheless, the observed benefits are supported by physiological and molecular data, as discussed below.

Supporting these findings, mechanistic studies provide evidence that TNF-α inhibitors may improve surrogate markers of cardiovascular health [33-36]. In a prospective study of 29 patients with moderate-to-severe psoriasis, researchers found that both endothelial function (assessed by flow-mediated endothelial-dependent vasodilation) and carotid arterial stiffness (measured by pulse wave velocity) improved from baseline after a six-month course of adalimumab (a widely used TNF-α inhibitor) [34]. Furthermore, in a larger study, the PROGRESS trial in North America showed that, among patients with chronic plaque psoriasis, CRP levels decreased during adalimumab therapy in those with suboptimal responses to prior therapies, including methotrexate and phototherapy [37]. Moreover, studies have shown that adalimumab decreases E-selectin (a marker of endothelial activation and dysfunction) and IL-22 (a cytokine implicated in psoriasis-associated inflammation and atherosclerosis) [38]. Collectively, these findings support a mechanistic rationale for the cardiovascular benefits of TNF-α inhibition; however, given that these studies assess biomarkers without direct correlation with clinical cardiovascular outcomes, the true clinical significance of these improvements remains to be confirmed in long-term outcome studies.

Despite broad evidence supporting cardiovascular benefit, some studies report inconsistent results. Although TNF-α inhibition is expected to improve glucose metabolism given its role in insulin resistance, clinical findings remain mixed. Some studies report improved insulin sensitivity and glycemic control, whereas others report neutral or adverse effects [1,39,40]. These inconsistencies may stem from short follow-up durations, inadequate adjustment for lifestyle or concurrent medication effects, and variable changes in body weight. Weight gain has been observed with TNF-α inhibitors, possibly reflecting improved disease control, restoration of appetite, or normalization of metabolic rate, which could offset cardiovascular benefits [1,41].

Furthermore, differences among TNF-α inhibitors may also contribute. Although adalimumab, etanercept, and infliximab all target TNF-α, they differ in molecular structure, pharmacokinetics, and binding affinity, potentially leading to variable cardiovascular outcomes [7]. Patient heterogeneity, including disease duration and baseline cardiovascular risk, further complicates interpretation. For example, a study of infliximab in psoriasis demonstrated improvement in skin disease but inconsistent effects on left ventricular remodeling, with mixed changes in ventricular mass and wall thickness [42]. These findings underscore the challenge of interpreting surrogate cardiac markers, which remain poorly validated against definitive cardiovascular outcomes in psoriatic populations.

TNF-α Inhibitors and HS

HS is a chronic inflammatory disease strongly associated with increased cardiovascular risk [43,44]. Patients with HS exhibit higher rates of obesity, diabetes, dyslipidemia, hypertension, and metabolic syndrome [44]. Obesity itself promotes systemic inflammation through TNF-α-mediated effects on insulin signaling and adipose tissue cytokine expression, contributing to a 21% higher risk of myocardial infarction, a 22% higher risk of cerebrovascular accident, and a 60% higher risk of cardiovascular death [4]. By inhibiting TNF-α signaling, biologic therapies may attenuate this inflammatory cascade, improve metabolic parameters, and potentially reduce cardiovascular risk.

A prospective case-control study of 19 HS patients treated with adalimumab demonstrated significantly elevated baseline levels of IL-6, IL-8, IL-10, IL-17A, soluble TNF receptor II, CRP, and ESR compared with healthy controls [45]. Following treatment, all markers decreased in parallel with clinical improvement, indicating suppression of systemic inflammation. These biomarkers are well-established mediators of cardiovascular risk. For example, IL-6 regulates lipid metabolism and promotes atherogenesis, while TNF-α contributes to microvascular dysfunction, insulin resistance, and endothelial activation [1,45,46]. These findings are supported by more robust data from the PIONEER I and II RCTs, which evaluated adalimumab in more than 600 patients with moderate-to-severe HS over 36 weeks [4]. After loading, regular adalimumab significantly reduced established pro-inflammatory and pro-thrombotic indicators, including the systemic immune inflammation index, neutrophil-to-lymphocyte ratio, platelet-to-lymphocyte ratio, and monocyte-to-lymphocyte ratio. All ratios were reduced from baseline, with sustained responses. Patients switched to a placebo experienced reversal of these changes, underscoring a treatment-dependent effect [4]. The correlation between reductions in inflammatory markers and disease severity suggests that systemic inflammatory control in HS may translate into meaningful cardiovascular benefit.

Although these findings are promising, none of the available studies correlated reductions in inflammatory mediators with hard cardiovascular endpoints, limiting conclusions about true clinical benefit [4,44,45]. While improvements in biomarkers suggest potential cardiovascular protection, whether these translate into reduced MACE (as observed in psoriasis with TNF-α inhibitors) remains unproven. Additionally, the RCTs cited above were primarily designed to evaluate cutaneous efficacy rather than cardiovascular outcomes, with most analyses conducted post hoc [4]. This limits causal interpretation and raises concerns about potential data overextension. Furthermore, although the post hoc analysis included long-term follow-up in the open-label extension up to 144 weeks, the duration and sample sizes remain insufficient to establish definitive long-term cardiovascular safety or benefit [4].

TNF-α Inhibitors and HF: A Special Safety Consideration

While TNF-α inhibitors are generally considered cardioprotective or neutral with respect to cardiovascular outcomes, isolated evidence suggests potential adverse effects in specific contexts. Notably, high-dose infliximab and etanercept have been associated with worsening HF in patients with pre-existing moderate-to-severe HF [47,48]. In the ATTACH trial (a randomized, double-blind, placebo-controlled pilot trial of infliximab), patients receiving infliximab 10 mg/kg showed a statistically significant increase in death or hospitalization for worsening HF compared with both placebo groups and patients receiving 5 mg/kg infliximab [47]. This has prompted changes to guidelines, with the European Society of Cardiology recommending cautious use for patients with HF [49]. The mechanism likely reflects TNF-α’s complex role in myocardial remodeling and contractility [42]. Outside HF-specific considerations, there is currently no substantial evidence of an increased risk of other adverse cardiovascular outcomes associated with TNF-α inhibitor use in dermatological populations.

IL-12/23 inhibitor (ustekinumab)

Ustekinumab, a monoclonal antibody targeting the shared p40 subunit of IL-12 and IL-23, is an earlier-generation biologic that modulates both Th1 and Th17 inflammatory pathways [50,51]. Given the involvement of IL-12 and IL-23 in atherogenesis, dual inhibition may provide a cardioprotective mechanism [52,53]. Ustekinumab is discussed here as the sole representative of IL-12/23 inhibitors, as newer agents selectively target the IL-23 p19 subunit and are therefore considered separately under IL-23-specific therapies [11].

Pooled safety analyses from phase two and three trials have shown low and comparable rates of MACE between ustekinumab and placebo, supporting its cardiovascular safety but not demonstrating a clear risk reduction, as seen with TNF-α inhibitors [54]. Cohort studies support this. A nationwide Korean cohort study found no significant difference in MACE risk between ustekinumab and TNF-α inhibitors, although residual confounding and selection bias cannot be excluded [55]. In a larger cohort study comparing ustekinumab to three other biologics, a Swedish national registry study found no evidence of reduced MACE risk but confirmed long-term cardiovascular safety over more than 10,000 patient-years of follow-up [56]. Overall, while ustekinumab appears safe with respect to cardiovascular outcomes, current evidence does not support a protective effect. Unlike TNF-α inhibitors, ustekinumab carries no warning for HF, reinforcing its favorable long-term safety profile [57].

At the molecular level, several studies have demonstrated that ustekinumab reduces inflammatory mediators implicated in both psoriasis and cardiovascular disease. One study compared circulating protein levels of a predetermined cardiovascular protein panel in ten patients with psoriasis before and after 12 weeks of ustekinumab treatment against 18 healthy controls, revealing that out of the 25 proteins that were elevated at baseline relative to controls, eight of these proteins were downregulated by ustekinumab to below the thresholds associated with increased cardiovascular risk. This included widely established pro-inflammatory mediators such as IL-6, E-selectin, and TNF receptor I [58]. This is supported by studies showing both reductions in inflammatory markers (e.g., TNF-α and IL-6) and improvements in coronary flow reserve, pulse wave velocity, and augmentation index (independent markers of cardiovascular risk). This study showed that these changes were specific to IL-12/23 inhibitors, which produced greater improvements in coronary, arterial, and myocardial function than cyclosporine and TNF-α inhibitors [59]. However, caution is warranted when interpreting these results, which were measured four months post-treatment. In a phase IV randomized, double-blind, placebo-controlled study (VIP-U Trial), 18F-FDG PET/CT was used to examine vascular inflammation in patients with psoriasis receiving ustekinumab and found significant reductions in aortic vascular inflammation compared with placebo over 12 weeks. However, despite an association with a sustained reduction in inflammatory cytokines at 52 weeks, the improvement in aortic vascular inflammation was not maintained at this time, indicating a transient effect [60]. This reflects a key issue with short trial duration and suggests that the inflammatory pathways modulated by ustekinumab may not be the principal drivers of cardiovascular risk in psoriasis, underscoring the complexity of immune-cardiovascular interactions.

IL-17 inhibitors

IL-17 inhibitors are a new class of biologics that target the IL-17 pathway, which is central to psoriatic inflammation [61,62]. IL-17A promotes keratinocyte proliferation, neutrophil recruitment, and pro-inflammatory cytokine release, and contributes to atherosclerosis through mechanisms including endothelial dysfunction, vascular inflammation, and plaque instability [63,64]. Consequently, the cardiovascular effects of IL-17 inhibition remain an active area of investigation.

Although the evidence base for this class is less mature than for earlier biologics, emerging data indicate favorable cardiovascular safety [10]. Unlike TNF-α inhibitors, IL-17 inhibitors have not been associated with HF exacerbation. Interestingly, secukinumab has shown potential cardioprotective effects in myocarditis, with case series reporting that IL-17A-associated myocarditis resolved following treatment with secukinumab [65]. This suggests a potential therapeutic role for IL-17 blockade in cardiac inflammation, although further studies are needed to confirm its prevalence and clinical relevance.

Beyond myocarditis, the cardiovascular effects of IL-17 inhibitors appear neutral [66]. Pooled safety analyses from large RCTs (UNCOVER-1, UNCOVER-2, and UNCOVER-3 for ixekizumab; ERASURE and FIXTURE for secukinumab) have reported low and comparable rates of MACE between IL-17 inhibitors and placebo over short-term follow-up (three to five years) [61,66]. However, given the relatively recent introduction of this class, long-term cardiovascular outcomes remain unknown.

To investigate the mechanisms by which IL-17 inhibitors may affect cardiovascular health, the ObePso study examined the molecular link between psoriasis and adipose tissue inflammation, demonstrating that obese psoriasis patients had elevated adipose tissue inflammation that was attenuated following secukinumab treatment [67]. As adipose inflammation contributes to metabolic syndrome and cardiovascular risk, these findings suggest an indirect cardiometabolic benefit through improved metabolic regulation. Consistent with this, secukinumab has shown modest reductions in CRP across multiple studies, although statistical significance was not consistently achieved, particularly at lower doses [68]. In contrast, ixekizumab has demonstrated more consistent CRP results, with the SPIRIT-P1 trial showing ixekizumab reduces CRP in patients with psoriatic arthritis, producing a substantially greater decrease in CRP-based disease activity (DAS28-CRP) compared with placebo over 24 weeks [69].

Further mechanistic studies have highlighted the importance of long-term follow-up in patients receiving biologics such as IL-17 inhibitors. Physiological studies investigating functional changes have shown that, if present, improvements may be delayed. In the VIP-S trial (a randomized placebo-controlled trial parallel to the VIP-U trial discussed earlier), 18F-FDG PET/CT imaging found a non-statistically significant reduction in aortic vascular inflammation in patients with moderate-to-severe psoriasis, either at 12 weeks or 52 weeks after 52 weeks of secukinumab therapy [70]. The effect is essentially neutral despite a significant improvement in skin disease activity. In contrast, in the 52-week randomized, double-blind, placebo-controlled CARIMA trial, although the authors found that psoriasis patients receiving secukinumab had increased flow-mediated dilation (FMD) vs placebo after 12 weeks, this difference did not reach statistical significance until repeat measurement at week 52, indicating that IL-17 blockade may have delayed cardiovascular protective effects [71]. This is a significant limitation of many studies investigating the cardiovascular effects of secukinumab in dermatological patients, particularly given that, in the latter trial, the absolute increase in FMD was clinically meaningful, with a corresponding 13% reduction in relative cardiovascular risk [71]. Further studies with long-term follow-up, coupled with reporting of cardiovascular endpoints, would provide additional information.

IL-23 inhibitors

IL-23 inhibitors are one of the newest classes of biologics for psoriasis, selectively targeting the IL-23 pathway while sparing IL-12 [11,72]. IL-23 is central to Th17 cell differentiation and maintenance, driving IL-17 production and sustaining psoriatic inflammation [23]. It has also been implicated in atherosclerosis through pro-inflammatory macrophage polarization and plaque instability [52]. Selective IL-23 inhibition with agents such as guselkumab, tildrakizumab, and risankizumab, therefore, offers targeted anti-inflammatory effects with potentially fewer off-target effects than dual IL-12/23 blockade with ustekinumab [11,73,74].

To date, studies investigating the cardiometabolic safety of IL-23 inhibitors in dermatological patients have consistently demonstrated no evidence of increased risk of HF [72,75]. Pooled analyses from large RCTs, including those evaluating tildrakizumab in moderate-to-severe psoriasis, report low and comparable rates of cardiovascular events between treatment and placebo groups, suggesting short-term cardiovascular neutrality [72]. However, follow-up durations of up to three years and low event rates limit conclusions regarding long-term cardiovascular benefit or harm. As with IL-17 inhibitors, current data are derived mainly from RCTs designed for cutaneous efficacy rather than cardiovascular outcomes, underscoring the need for longer-term and purpose-built studies to clarify their cardiovascular impact. This is underscored by findings from multiple long-term pharmacovigilance analyses reporting safety concerns regarding cardiovascular outcomes associated with the commonly used IL-23 inhibitor, risankizumab. A disproportionality analysis of the FDA Adverse Event Reporting System (FAERS) revealed that risankizumab was associated with disproportionately higher reporting of cerebrovascular accidents compared with other treatments for plaque psoriasis, including ixekizumab (IL-17 inhibitor), guselkumab (IL-23 inhibitor), and etanercept (TNF-α inhibitor) [76]. However, FAERS signals reflect spontaneous reporting and do not establish causality; their interpretation is limited by underreporting, reporting bias, and the lack of denominator data. As such, these findings should be interpreted as hypothesis-generating pharmacovigilance signals only and should not influence clinical decision-making outside the context of ongoing safety monitoring until confirmed by robust long-term observational or comparative studies.

From a mechanistic perspective, evidence for cardiovascular protection pathways mediated by IL-23 inhibitors is not well established and, if present, implicates higher-than-standard doses in selected high-risk subgroups to produce cardiometabolic benefits. Initial data from post hoc cardiometabolic analyses of the reSURFACE one and two trials showed no clinically significant changes in body mass index, blood pressure, fasting glucose, or lipid measures in patients receiving tildrakizumab for moderate-to-severe plaque psoriasis, regardless of dose (100 mg or 200 mg). This was true at 64 weeks for reSURFACE 1 and 52 weeks for reSURFACE 2, irrespective of metabolic syndrome status [77]. In contrast, a prospective 2025 study of patients with psoriasis and coexisting metabolic syndrome who had an inadequate response to tildrakizumab 100 mg found that escalating to 200 mg resulted in significant reductions in total cholesterol, LDL cholesterol, and fasting glucose. In contrast, the standard 100 mg dose produced no meaningful metabolic change [73]. Collectively, these findings indicate that, although tildrakizumab is metabolically neutral in most patients, a dose-dependent improvement in lipid and glycemic parameters may occur in partial responders with metabolic syndrome, potentially reflecting the need for greater IL-23 pathway suppression to achieve metabolic benefit in this high-risk subgroup. The relevance of these findings to the pharmacovigilance reports highlighted above, and whether these cardiometabolic changes translate into consequential changes in MACE, remains to be determined.

IL-23 Inhibitors vs IL-12/23 Inhibitors

A key mechanistic distinction between ustekinumab (an IL-12/23 inhibitor) and selective IL-23 inhibitors (guselkumab, tildrakizumab, and risankizumab) lies in their blockade of IL-12. IL-12 drives Th1 responses and interferon-γ production, both of which are implicated in atherosclerosis [52]. Theoretically, dual IL-12/23 inhibition could confer broader anti-atherogenic effects than selective IL-23 inhibition. However, current evidence remains inconclusive, and no significant direct comparative cardiovascular outcome data are yet available.

IL-4/IL-13 inhibitors (dupilumab)

Dupilumab, a monoclonal antibody that blocks the IL-4 receptor α subunit and inhibits IL-4 and IL-13 signaling, is the only widely established IL-4/IL-13 inhibitor in clinical practice with sufficient evidence to support cardiovascular evaluation [78]. It is primarily approved for the treatment of AD, asthma, and chronic rhinosinusitis with nasal polyposis [79,80]. While AD has been less consistently linked to cardiovascular disease than psoriasis or HS, emerging evidence suggests increased cardiovascular risk in severe cases, prompting interest in dupilumab’s systemic effects [81,82].

Large multinational cohort studies report no increased risk of MACE with dupilumab compared with other therapies, and evidence suggests that it may reduce cardiovascular risk. In a global retrospective cohort study investigating cardiovascular and metabolic outcomes in AD patients receiving dupilumab vs methotrexate, the study found that dupilumab was associated with a decreased risk of peripheral vascular disease, deep vein thrombosis, hypertension, type 2 diabetes mellitus (T2DM), and obesity within the first year of treatment [15]. The authors also conducted a second analysis comparing dupilumab with cyclosporine in AD. They found that patients given dupilumab had a reduced risk of hypertension, hyperlipidemia, and T2DM within one year. Crucially, in the subsequent analysis, these changes were statistically significant at three years. Collectively, these findings suggest a favorable cardiometabolic safety profile, supported by mechanistic studies demonstrating reduced systemic and aortic inflammation following dupilumab treatment [83] and improved endothelial barrier function through reduced vascular leakage into inflamed skin [84]. However, improvements in metabolic diagnoses and surrogate cardiometabolic markers should not be interpreted as evidence of reduced MACE. The available data remain limited by retrospective study design, potential residual confounding, and reliance on surrogate endpoints. Overall, dupilumab appears cardiometabolically safe; however, long-term prospective studies in higher-risk populations incorporating hard cardiovascular outcomes are required to determine its true impact on MACE.

Study limitations and methodological considerations

Across the studies reviewed, several methodological limitations restrict the strength and generalizability of current evidence. Marked heterogeneity in study design, populations, follow-up durations, and outcome definitions, including MACE, impedes direct comparison and synthesis [9,28]. Many studies rely on surrogate cardiovascular markers, such as inflammatory biomarkers or vascular imaging, rather than on clinical endpoints, including myocardial infarction or stroke [34,39,40,42,60,70]. Such studies are also usually restricted to treatment responders, introducing selection bias and limiting generalizability [34,39,42,60,70]. Therefore, although they provide valuable insights, their correlation with long-term outcomes in dermatological populations remains uncertain.

Additionally, although RCTs are methodologically robust, they are often short and underpowered to detect rare cardiovascular events [10,72]. Conversely, large registry and cohort studies provide real-world relevance but remain prone to residual confounding, including unadjusted differences in disease severity, comorbidities, treatment duration, and traditional cardiovascular risk factors [12]. Publication bias further limits interpretation, as studies demonstrating favorable cardiovascular effects are more likely to be published, potentially inflating perceived benefit [60,70]. This is compounded in meta-analyses, where limited event numbers and selective safety reporting may distort pooled outcomes [85].

Finally, evidence for newer biologics remains sparse and occasionally contradictory, reflecting short follow-up periods and limited event capture [73,77]. Collectively, these constraints underscore the need for long-term, adequately powered prospective studies with cardiovascular outcomes as primary endpoints to better define the true cardiometabolic impact of biologic therapy in dermatologic disease.

Gaps in knowledge and future studies

Despite an expanding evidence base, significant uncertainties remain regarding the cardiovascular effects of biologic therapy in dermatologic disease. Long-term prospective data beyond 5-10 years are scarce, limiting understanding of sustained cardiovascular safety and potential protective effects, particularly for IL-17, IL-23, and IL-4/IL-13 inhibitors [15,66,72]. The absence of head-to-head comparative trials across biologic classes further constrains evidence-based treatment selection for patients with elevated cardiovascular risk.

Data on subclinical atherosclerosis with newer biologics are limited, with few studies evaluating arterial stiffness or endothelial function [83,84,86]. Similarly, the validity of surrogate endpoints such as inflammatory biomarkers and vascular imaging requires confirmation against hard cardiovascular outcomes [60,70]. The optimal treatment duration and persistence of cardiovascular benefit after biologic discontinuation also remain unknown. In AD, the cardiovascular impact of dupilumab remains underexplored despite increasing recognition of cardiometabolic risk in severe disease [81].

Future research should prioritize long-term, adequately powered RCTs and prospective cohort studies with extended follow-up to capture late cardiovascular events and to define the true risk-benefit profile of biologics. These ought to be directly correlated with mechanistic studies, biomarker profiling, vascular imaging, and molecular analyses. Furthermore, robust adjustment for comorbidities and detailed clinical phenotyping will be essential to isolate drug effects from underlying disease activity. Disease-specific and dose-response studies, alongside exploration of potential synergy between biologics and conventional cardiovascular therapies, are also warranted.

Clinical implications

Although definitive cardiovascular outcome trials are awaited, several practical considerations emerge from current evidence. Cardiovascular risk assessment should be routine in patients with moderate-to-severe inflammatory skin disease to inform treatment selection. TNF-α inhibitors, which have the most robust cardiovascular benefits [31], may be preferred in patients with high cardiovascular risk, though they remain contraindicated in moderate-to-severe HF [47,48]. This should be achieved through coordinated, multidisciplinary care involving dermatology, cardiology, and rheumatology, thereby minimizing risk and optimizing outcomes. Patients should also be educated about the potential cardiovascular benefits of biologics while reinforcing conventional preventive measures such as smoking cessation, diet, exercise, and statin use when indicated [32]. Together with regular monitoring of metabolic parameters, this will help improve patient outcomes and provide further insights into the cardiovascular outcomes of biologic therapy in dermatological disease.

## Conclusions

Biologic therapies, particularly TNF-α inhibitors, demonstrate potential cardiovascular benefit in inflammatory dermatologic diseases. However, results across classes remain inconsistent, reflecting heterogeneity in study design and limited long-term follow-up, particularly for newer classes. Mechanistic and clinical evidence suggest that systemic inflammation control may underpin cardiovascular improvement, yet this effect is unlikely to be uniform across biologic classes.

Personalized treatment selection, integrated cardiovascular risk assessment, and multidisciplinary management are essential to optimize outcomes. Ultimately, adequately powered, long-term studies with cardiovascular endpoints are needed to confirm sustained benefit, refine patient selection, and inform treatment duration. Biologic therapy thus holds promise not only for effective control of skin disease but also for cardiovascular risk modification in systemic inflammatory disease.

## Appendices

This supplementary file contains the extended study characteristics table (Table 5), which supports the findings of this review, and the PRISMA 2020 checklist (Table 6).

**Table 5:** Extended study characteristics table Characteristics of the 74 included studies grouped by biologic class, showing the range of study designs, populations, and cardiovascular outcomes assessed. CV: cardiovascular, MACE: major adverse cardiovascular events, MI: myocardial infarction, HF: heart failure, PWV: pulse wave velocity, IMT: intima–media thickness, PET/CT: positron emission tomography/computed tomography, DAS28-CRP: Disease Activity Score based on 28-joint count and C-reactive protein, T2DM: type 2 diabetes mellitus, AD: atopic dermatitis, HS: hidradenitis suppurativa

Biologic class	Predominant dermatologic indication(s)	Main study designs represented	Typical sample size range	Typical follow-up	Cardiovascular outcomes reported	Evidence volume (qualitative)	Overall evidence profile
TNF-α inhibitors (adalimumab, etanercept, infliximab, others)	Psoriasis, psoriatic arthritis, HS	Population-based cohorts, RCTs, post-hoc analyses of phase III trials, and mechanistic vascular/biomarker studies	From small mechanistic cohorts (<50) to large registries (>10,000 patients)	6–24 months in mechanistic and phase IV trials; up to ≥10 years in registry cohorts	MACE (MI, stroke, CV death), heart failure, CRP and other inflammatory markers, arterial stiffness (PWV, augmentation index), carotid IMT	High (the largest and most mature dataset)	Evidence of cardioprotection in psoriasis (reduced MACE compared with methotrexate and phototherapy), consistent with improvements in CRP and vascular biomarkers; however, high-dose infliximab and etanercept are associated with worsening heart failure in patients with pre-existing moderate–severe HF.
IL-12/23 inhibitor (ustekinumab)	Psoriasis, psoriatic arthritis	Phase II–III RCTs, pooled safety analyses, nationwide registry cohorts, mechanistic PET/CT, and vascular function studies	Small mechanistic cohorts (~10–50 patients) to national registry datasets (>5,000 patients; >10,000 patient-years)	12–24 weeks in mechanistic and phase IV studies; several years in observational registry data	MACE, all-cause mortality, aortic vascular inflammation (PET/CT), coronary flow reserve, arterial stiffness indices, circulating cardiovascular protein panels	Moderate	Consistent cardiovascular safety with no excess MACE vs placebo or TNF-α inhibitors; mechanistic data show transient reductions in vascular inflammation and improvements in myocardial/vascular function, but no clear long-term MACE risk reduction is demonstrated.
IL-17 inhibitors (secukinumab, ixekizumab)	Psoriasis, psoriatic arthritis	Phase III RCTs (e.g., UNCOVER, ERASURE/FIXTURE, FUTURE, SPIRIT), pooled safety analyses, mechanistic PET/CT and FMD studies, adipose-tissue inflammation studies, case series (myocarditis)	Moderate to large RCTs (hundreds to a few thousand participants); small mechanistic cohorts	24–52 weeks in most trials; pooled safety follow-up up to ~5 years	MACE, CRP, DAS28-CRP, aortic vascular inflammation (PET/CT), FMD, adipose-tissue inflammation	Moderate/emerging	Overall, the cardiovascular profile appears neutral, with low and comparable MACE rates relative to placebo; modest or inconsistent reductions in CRP; limited or delayed effects on vascular function; and case series suggesting potential benefit in IL-17A-associated myocarditis; however, long-term outcome data are lacking.
IL-23 inhibitors (guselkumab, tildrakizumab, risankizumab)	Psoriasis, psoriatic arthritis, metabolic syndrome subgroups	Phase III RCTs (e.g., reSURFACE, VOYAGE, DISCOVER), post-hoc cardiometabolic analyses, and small prospective metabolic studies	RCT and post-hoc populations typically in the hundreds to low thousands; smaller targeted cohorts in high-risk patients	24–52 weeks in pivotal trials; some extension/safety datasets up to ~3 years	MACE (from pooled safety datasets), CRP, IL-6, neutrophil-to-lymphocyte, platelet-to-lymphocyte, and monocyte-to-lymphocyte ratios, body weight, waist circumference, blood pressure, fasting glucose, lipid profile	Emerging	Short- to medium-term safety appears reassuring with low MACE rates and broadly neutral cardiovascular signal. Post hoc data suggest improvements in systemic inflammatory indices and, at higher doses and in metabolic syndrome subgroups, favourable changes in lipids and glycaemic parameters. A potential pharmacovigilance signal for cerebrovascular events with risankizumab warrants cautious interpretation pending long-term observational data.
IL-4/IL-13 inhibitor (dupilumab)	AD (with/without atopic comorbidities); background evidence in asthma and chronic rhinosinusitis with nasal polyposis	Large multinational cohort studies, phase III RCTs, small mechanistic PET/CT, and endothelial-barrier studies	Small mechanistic cohorts (~10–30 patients) to large administrative/cohort datasets (thousands of patients)	2–4 years in cohort studies; weeks to months in mechanistic and imaging studies	MACE, broader cardiometabolic safety endpoints, systemic and aortic inflammation (PET/CT), endothelial barrier function, and vascular leakage	Moderate/emerging	Available evidence indicates a favourable cardiometabolic safety profile, with no increased risk of MACE compared with conventional systemic therapies; recent cohort data suggest a reduced risk of hypertension, dyslipidaemia, T2DM, and obesity compared with methotrexate or cyclosporine. Mechanistic data demonstrate reduced systemic and vascular inflammation and improved endothelial barrier integrity, although confirmation in higher-risk populations with longer follow-up is needed.

**Table 6:** PRISMA 2020 checklist The PRISMA 2020 checklist summarizes how each reporting item of the PRISMA 2020 framework is addressed within the manuscript, including the corresponding section. Items marked as not applicable reflect methodological features of this review, such as the absence of a registered protocol, meta-analysis, or quantitative synthesis. PRISMA: Preferred Reporting Items for Systematic Reviews and Meta-Analyses

Section and topic	Item #	Checklist item	Location where the item is reported
TITLE			
Title	1	Identify the report as a systematic review.	Title
ABSTRACT			
Abstract	2	See the PRISMA 2020 for the abstracts checklist.	Abstract
INTRODUCTION			
Rationale	3	Describe the rationale for the review in the context of existing knowledge.	Introduction
Objectives	4	Provide an explicit statement of the objective(s) or question(s) the review addresses.	Introduction
METHODS			
Eligibility criteria	5	Specify the inclusion and exclusion criteria for the review and how studies were grouped for the syntheses.	Review
Information sources	6	Specify all databases, registers, websites, organisations, reference lists, and other sources searched or consulted to identify studies. Specify the date when each source was last searched or consulted.	Review
Search strategy	7	Present the full search strategies for all databases, registers, and websites, including any filters and limits used.	Review
Selection process	8	Specify the methods used to decide whether a study met the inclusion criteria of the review, including how many reviewers screened each record and each report retrieved, whether they worked independently, and, if applicable, details of automation tools used in the process.	Review
Data collection process	9	Specify the methods used to collect data from reports, including how many reviewers collected data from each report, whether they worked independently, any processes for obtaining or confirming data from study investigators, and, if applicable, details of automation tools used in the process.	Review
Data items	10a	List and define all outcomes for which data were sought. Specify whether all results that were compatible with each outcome domain in each study were sought (e.g., for all measures, time points, analyses), and if not, the methods used to decide which results to collect.	Review
	10b	List and define all other variables for which data were sought (e.g., participant and intervention characteristics, funding sources). Describe any assumptions made about any missing or unclear information.	Review
Study risk of bias assessment	11	Specify the methods used to assess risk of bias in the included studies, including details of the tool(s) used, how many reviewers assessed each study, and whether they worked independently, and if applicable, details of automation tools used in the process.	Review
Effect measures	12	Specify for each outcome the effect measure(s) (e.g., risk ratio, mean difference) used in the synthesis or presentation of results.	Not applicable – this review used narrative synthesis only
Synthesis methods	13a	Describe the processes used to decide which studies were eligible for each synthesis (e.g., tabulating the study intervention characteristics and comparing against the planned groups for each synthesis (item #5)).	Review
	13b	Describe any methods required to prepare the data for presentation or synthesis, such as handling of missing summary statistics or data conversions.	Review
	13c	Describe any methods used to tabulate or visually display the results of individual studies and syntheses.	Not applicable – no meta-analysis was performed
	13d	Describe any methods used to synthesize results and provide a rationale for the choice(s). If meta-analysis was performed, describe the model(s), method(s) to identify the presence and extent of statistical heterogeneity, and software package(s) used.	Not applicable – no meta-analysis was performed
	13e	Describe any methods used to explore possible causes of heterogeneity among study results (e.g., subgroup analysis, meta-regression).	Not applicable – no meta-analysis was performed
	13f	Describe any sensitivity analyses conducted to assess the robustness of the synthesized results.	Not applicable – no meta-analysis was performed
Reporting bias assessment	14	Describe any methods used to assess the risk of bias due to missing results in a synthesis (arising from reporting biases).	Not applicable – no meta-analysis was performed
Certainty assessment	15	Describe any methods used to assess certainty (or confidence) in the body of evidence for an outcome.	Not applicable – no meta-analysis was performed
RESULTS			
Study selection	16a	Describe the results of the search and selection process, from the number of records identified in the search to the number of studies included in the review, ideally using a flow diagram.	Review
	16b	Cite studies that might appear to meet the inclusion criteria, but which were excluded, and explain why they were excluded.	Not reported
Study characteristics	17	Cite each included study and present its characteristics.	Review and discussion
Risk of bias in studies	18	Present assessments of risk of bias for each included study.	Review
Results of individual studies	19	For all outcomes, present, for each study: (a) summary statistics for each group (where appropriate) and (b) an effect estimate and its precision (e.g., confidence/credible interval), ideally using structured tables or plots.	Not reported
Results of syntheses	20a	For each synthesis, briefly summarize the characteristics and risk of bias among contributing studies.	Review and discussion
	20b	Present the results of all statistical syntheses conducted. If a meta-analysis was conducted, present, for each, the summary estimate and its precision (e.g., confidence/credible interval), and measures of statistical heterogeneity. If comparing groups, describe the direction of the effect.	Review and discussion
	20c	Present the results of all investigations of possible causes of heterogeneity among study results.	Review and discussion
	20d	Present the results of all sensitivity analyses conducted to assess the robustness of the synthesized results.	Review and discussion
Reporting biases	21	Present assessments of risk of bias due to missing results (arising from reporting biases) for each synthesis assessed.	Review
Certainty of evidence	22	Present assessments of certainty (or confidence) in the body of evidence for each outcome assessed.	Review and discussion
DISCUSSION			
Discussion	23a	Provide a general interpretation of the results in the context of other evidence.	Discussion
	23b	Discuss any limitations of the evidence included in the review.	Limitations
	23c	Discuss any limitations of the review processes used.	Limitations
	23d	Discuss implications of the results for practice, policy, and future research.	Limitations
OTHER INFORMATION			
Registration and protocol	24a	Provide registration information for the review, including the register name and registration number, or state that the review was not registered.	Not registered
	24b	Indicate where the review protocol can be accessed, or state that a protocol was not prepared.	Protocol was not prepared
	24c	Describe and explain any amendments to information provided at registration or in the protocol.	Not applicable
Support	25	Describe sources of financial or non-financial support for the review, and the role of the funders or sponsors in the review.	Review
Competing interests	26	Declare any competing interests of review authors.	Review
Availability of data, code, and other materials	27	Report which of the following are publicly available and where they can be found: template data collection forms; data extracted from included studies; data used for all analyses; analytic code; any other materials used in the review.	Not reported
